# 

**Published:** 1985-01

**Authors:** 


					JANUARY 1985
VOLUME 100 (i)
NUMBER 373
Published Quarterly
Annual Subscription ?10 Post Free
BRISTOL
MEDICO
CHIRTJRGICAL
KXJKNAL
(Established 1883i
BRISTOL
MEDICO
CHIRURGICAL
XXJRNAL
Incorporating the Medical Journal of the South West
Printed in Great Britain by John Wright & Sons (Printing) Ltd, at
The Stonebridge Press, Bristol
Editorial Committee
EDITOR
Mr. M. G. Wilson
40 Coombe Lane, Bristol BS92BJ
MEMBERS
Dr. M. B. Lennard
Dr. D. W. Wright
The Hon. Treasurer of the Society
The Hon. Secretary of the Society
SECRETARY
Mr. B. P. Jones, The Library, Medical School,
University Walk, Bristol BS8 7 TD
Correspondents
Dr. J. D. Da vies
Dr. J. Jancar
Dr. H. G. Mather
Professor G. M. Stirrat
Dr. J. L. G. Thomson
Dr. M. J. Whitfield
Professor R. C. N. Williamson
Dr. D. W. Wright
Bristol Medico-Chirurgical Society
PRESIDENT
Dr /. S. Bailey
PRESIDENT ELECT
Dr J. Jancar
HONORARY SECRETARY
Dr. H. M. Norman
Hillside, Vicarage Lane, Barrow Gurney
HONORARY TREASURER
Dr. J. B. Penry.
Southmead Hospital, Bristol BS10 5NB
The Society was founded in 1874. In 1883 publica-
tion of the Journal began and has continued quar-
terly ever since then. During the years 1 949 to 1 962
it appeared under the title of 'The Medical Journal of
the South West'.
Notice to Contributors
The Journal is especially concerned to provide a place for
the recording of medical thought, interests and practice in
and around Bristol. It therefore welcomes articles that, as
well as being of immediate interest to members, will
document the local and contemporary medical scene for
our successors.
Original articles are preferred in the form proposed by the
International Committee of Medical Editors (The
Vancouver System) and published as an article in the
British Medical Journal in 1979 (Br. Med. J. 1, 532-535).
This has been reprinted as a pamphlet and may be obtained
from the Editor of the B.M.J., B.M.A. House, Tavistock
Square, London WC1H 9JR (enclose 50p).
Also welcome are letters to the Editor, Book Reviews,
news of important appointments and events, etc.

				

